# Herpes simplex viral encephalitis with acute memory impairment and low cellular cerebrospinal fluid: A case report with systematic review literature

**DOI:** 10.1016/j.idcr.2024.e01981

**Published:** 2024-05-18

**Authors:** Mostafa Mahmoud Meshref, Abdullah Ashraf Hamad, Amira Mohammed Taha, Yahia Nabil, Ahmed Hassan, Ahmed Samir Farw, Mohamed Elmasry, Abdulqadir J. Nashwan

**Affiliations:** a Neurology Department, Faculty of Medicine, Al-Azhar University, Cairo, Egypt; b Faculty of Medicine, Menoufia University, Menoufia, Egypt; c Faculty of Medicine, Fayoum University, Fayoum, Egypt; d Faculty of Medicine, Zagazig University, Egypt; e Neurology Department, Tawam Hospital, Al-Ain, United Arab Emirates; f Alexandria Medical School, Alexandria University, Egypt; g Hamad Medical Corporation (HMC), Doha, Qatar

**Keywords:** Herpes simplex virus, Polymerase chain reaction, Cerebrospinal fluid, Memory affection, Case report

## Abstract

Herpes simplex encephalitis (HSVE) is a potentially fatal infectious central nervous system (CNS) disorder. Thus, early detection is critical in determining the case's fate. Clinical history and examination, brain computed tomography, dynamic contrast-enhanced magnetic resonance imaging (DCE-MRI), and lumbar puncture have been used to establish a diagnosis. This report describes a case of HSVE with hypocellular cerebrospinal fluid (CSF) and an uncommon form of memory impairment. However, MRI results were consistent with HSVE, and CSF PCR tested positive for HSV-1 DNA that responded to treatment. We routinely advise patients to begin antiviral therapy as soon as possible to avoid complications.

## Highlights

• Herpes simplex encephalitis (HSVE) is a fatal CNS infection.
• Diagnosis of HSVE relies on clinical, imaging, and CSF analysis.
• Rare case: acute memory affection due to viral encephalitis with hypocellular CSF.
• Challenges in HSVE diagnosis: Symptom-imaging-CSF mismatch.
• Early antiviral treatment crucial for HSVE management.

## Introduction

Viral encephalitis is a severe neurological condition marked by inflammation of the brain parenchyma. The extent of brain involvement and prognosis is primarily determined by the causative pathogen and the host's immunological state [1]. Herpes simplex virus encephalitis (HSVE) is the most common viral encephalitis, with mortality rates up to 70 % in the lack of proper treatment, and only a minority retrieve their normal function [2]. Clinical findings, MRI scans, and the findings of CSF analysis are all crucial in diagnosing HSVE [3].

One or both temporal lobes are typically abnormal in herpes simplex encephalitis with occasional involvement of the orbitofrontal cortex, insula, or cingulate gyrus [3]. Temporal lobe systems are also responsible for remote memory and semantic memory (our facts and understanding of the world). This clarifies that memory loss is the most common and frequently disabling complication of viral encephalitis [4].

Damage to the temporal lobe in the dominant hemisphere is associated with semantic memory deficit and anomia. Simultaneously, lesions in the prefrontal cortex and subcortical areas that disrupt these areas' interconnections may produce executive dysfunction [5]. Moreover, it is well-known that involvement of the temporal lobe's cortex or hemorrhage, and the role of HSV as a differential diagnosis in rapid progressive dementia, are significant aspects to consider [6]. McGrath et al. reported that 69 % of patients with HSVE were presented with short- and long-term memory impairment, and 45 % were associated with personality and behavioral abnormalities [7]. Due to the variety and extent of cognitive sequelae, the knowledge of the impact of HSVE on acute cognitive dysfunction is based on detailed single case reports. Therefore, this report aimed to describe a case after HSVE where acute cognitive dysfunction were observed with MRI findings and hypocellular CSF. By presenting this particular case, we aim to contribute to the existing literature on viral encephalitis and deepen our comprehension of the various clinical and laboratory findings that can accompany this condition.

## Case report

A 46-year-old Egyptian male (working in Saudi Arabia) with no significant history of chronic medical disease presented with flu-like symptoms, including headache, malaise, and a low-grade fever (37.8 °C). Three days before he arrived at our hospital. One day later, the patient became distracted, and his wife noticed some memory concerns (primarily with random and recent memory). The patient was oriented on the day of arrival, with no signs of meningeal irritation and no fever (due to analgesic use). Furthermore, we discovered a decrease in attention span, which has been associated with memory affection. However, there was no irritability, agitation, fits, motor, or sensory deficit.

We ordered a computerized tomography (CT) of the brain, which revealed a normal brain with no mass, lesions, or vascular event. A lumbar puncture was also performed for CSF sampling, which revealed clear CSF with normal CSF opening pressure. Unexpectedly, we found hypocellular CSF (less than five cells) [3], [4] with predominant lymphocytes and occasional neutrophils with no red blood cells (RBCs). The CSF glucose level was 3.1 mmol/L (normal range: 2.2–3.9 mmol/L), and the CSF protein level was 56.5 mg/dl (normal range: 15–45 mg/dl). The white blood cell (WBC) count was 10.88 × 10^3^ on the complete blood count (CBC). After the patient's admission, CSF cultures were taken for bacteria and tuberculosis, and the results were negative. After that, we ordered a CSF polymerase chain reaction (PCR), through using Multiplex PCR techniques. The results were positive for herpes simplex virus (HSV) type 1 (HSV-1). Also, PCR panel for other viruses (including enteroviruses, CMV, and EBV) in the CSF samples were negative.

**Fig. 1:**
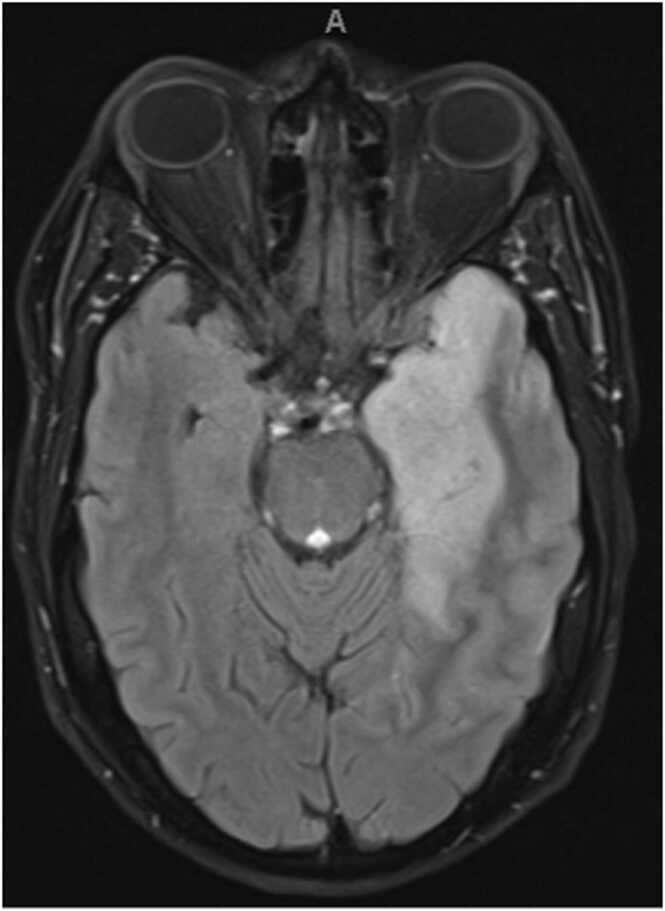
The dynamic contrast-enhanced magnetic resonance imaging (DCE-MRI) of patient’s brain.

Once the patient was admitted, we started empirical treatment immediately, including ceftriaxone 2 g IV BID (stopped after PCR results), acyclovir 1000 mg IV TID for 21 days, with good hydration and follow-up of renal function daily. Moreover, dexamethasone 8 mg IV QID was administered as an empirical treatment; however, it was decreased after a few days in a tapering manner. The dynamic contrast-enhanced magnetic resonance imaging (DCE-MRI) of his brain showed left mesial temporal, insular areas of abnormal signal with mass effect in the form of obliteration of the temporal horn and effacement of the cortical sulci with corresponding restricted diffusion (Fig. 1). However, no abnormal contrast enhancement was observed. After one month of follow-up, the patient showed a significant improvement after medical treatment, according to the patient's examination and family notes. Written informed consent was obtained from the patient for using the clinical images and the case details.

## Methods

We systematically reviewed case reports that reported herpes simplex encephalitis presenting or complicating with the acute onset of memory dysfunction. We searched PubMed, Scopus, and Web of Science from inception up to February 23, 2024, using the following search terms: (“herpes simplex” OR “HSV” OR “TORCH”) AND ("encephalitis" OR "brain infection") AND ("memory" OR "amnesia"). We excluded study designs other than case reports.

## Results

**Fig. 2:**
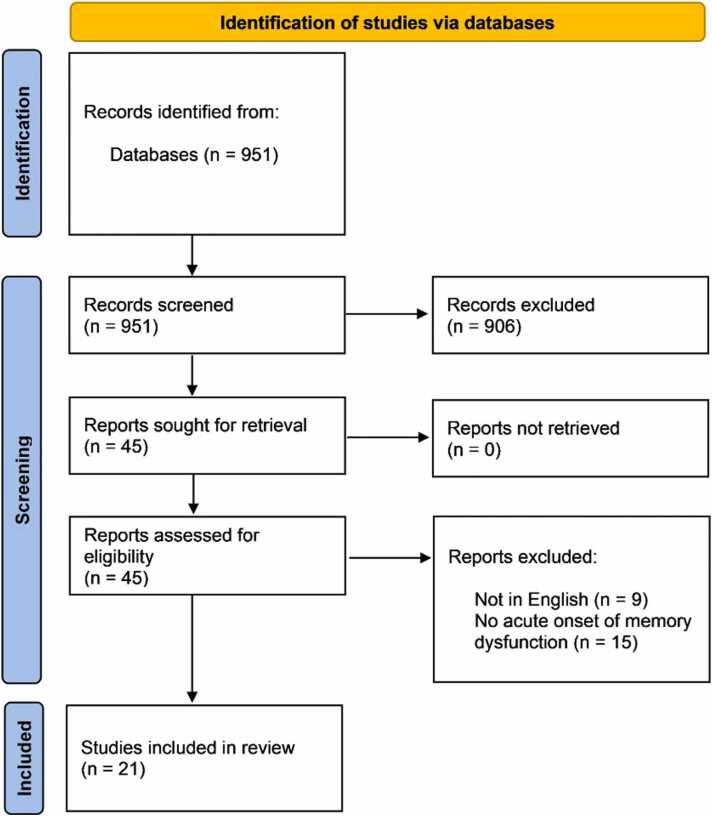
PRISMA chart.

Our search resulted in 951 citations. After screening the titles and abstracts of these articles, we identified 45 articles for full-text screening. Of these, nine articles were not in English, and 15 did not meet the inclusion criteria of reporting an acute onset of memory dysfunction. Finally, we included 21 articles [8], [9], [10], [11], [12], [13], [14], [15], [16], [17], [18], [19], [20], [21], [22], [23], [24], [25], [26], [27], [28] reporting 22 cases in this review. The age of the patients ranged from 13 to 78, and 7 cases [9], [10], [14], [18], [19], [22], [28] were females as shown in the PRISMA figure (Fig. 2**)**. All cases presented with or rapidly developed memory dysfunction, such as short-term memory impairment, retrograde or anterograde amnesia. All cases had favorable outcomes except two cases [8], [21] with fatal outcomes and four cases [11], [18], [21], [22] without reported outcomes. Table 1 displays the included cases' demographic characteristics, clinical features, and neuroimaging.

**Table 1:** Demographic characteristics, clinical features, and neuroimaging of the included cases.

Study	Age	Sex	Presenting features	CSF PCR for herpes virus	Neuroimaging	Treatment	Outcome
Grover 2004	NR	NR	Poor short-term memory and expressive dysphasia, present for several weeks	Not detected	Multiple foci of increased signal in periventricular and peripheral white matter [MRI], Generalized cortical atrophy [MRI]	Nil	Died of progressive Kaposi’s sarcoma. Necropsy identified HSV-1 in cerebral lesions
Borelli 2018	73	Female	Severe headache, confusion, and primarily anterograde amnesia	Positive	Left-predominant region of hyperintensity on the fluid-attenuated inversion recovery sequence in the anterior medial temporal lobe including the hippocampus and surrounding structures [MRI]	NR	Recovered, aside from residual short-term memory deficits
Chen 2023	58	Male	Short‐term memory deficits and absence seizures for 1 year	NR	hyperintense FLAIR lesions in the bilateral hippocampus and left insula lobe [MRI]. spikes and slow waves primarily in the left anterior temporal and sphenoidal regions [EEG].	Prednisone	The frequency of seizures reduced
Fujii 1999	51	Female	Generalized epileptic seizure and severe retrograde amnesia	Positive	abnormal high intensity areas in the bilateral medial temporal lobes, including the anterior two thirds of the hippocampal formation (hippocampus, dentate gyrus, and subiculum) and the posterior part of the amygdala [MRI]	NR	NR
Croll 2016	78	Male	Mild fever, fatigue, anorexia diarrhea, anxiety, depression and hand tremors. He had new-onset memory loss and confusion the following day.	Positive	Abnormal signal/patchy enhancement of the left temporal lobe indicative of encephalitis [MRI]	Acyclovir	He was discharged five days after admission
Geffen 2008	56	Male	Retrograde amnesia without consciousness disturbance	NR	EEG indicated mild abnormality. CT revealed shallow sulci, enlargement of ventricular system, and slight hemorrhage	Acyclovir, Fluconazole, Streptomycin, Rifampicin, Isoniazid, and antibiotics.	Discharged two months later
Soares-Ishigaki 2012	13	Male	Confusion, disconnected speech, migraine, fever and nuchal rigidity	Positive	MRI two years later showed areas of encephalomalacia predominantly involving the left temporal lobe, and to a lesser extent, the left frontal lobe, right temporal lobe, precuneus and lateral region of the left occipital lobe.	NR	Discharged one month later but with memory and cognitive impairment
Vedes 2012	43	Male	One-week history of confusion, memory loss with attention deficit, and strange behavior, being sometimes aggressive	Negative	CT showed a pattern of atrophy mainly involving the mesiotemporal region bilaterally, with focal enlargement of the lateral ventricles temporal horns. MRI showed an asymmetrical bilateral cortical and subcortical hyperintensity on T2- and fluid-attenuated-inversion-recovery- (FLAIR-) weighted signal in the mesiotemporal region and insula, more intense on the right side	Acyclovir and penicillin	The patient was discharged and continues follow-up
Wilson 1995	46	Male	Influenza-like illness with headache and fever. Memory of about 3-s duration	NR	CT scan indicated areas of low density particularly in the left temporal lobe extending into the inferior and posterior frontal lobe and into the right medial temporal lobe. MRI showed marked abnormality to be present in both hippocampal formations, both amygdalas, the substantia innominata on both sides, both mammillary bodies	NR	The patient was discharged and followed up
Eslinger 1993	17	Female	Flu-like symptoms and amnesia	NR	(Were performed 3 years later)	NR	Discharged 5 months later but was unable to smell or taste
Sellner 2009	25	Female	Was drowsy and with her tongue bitten. The patient had anterograde and retrograde amnesia and generalized seizure	Positive	MRI showed coronal FLAIR imaging showed T2 hyperintensities within the right temporopolar and temporomesial region	Aciclovir, lorazepam, and betamethasone	Discharged 4 weeks after initial hospitalization
Conlon 1988	27	Male	Sore throat, headache, malaise and fever. He then showed anterograde and retrograde amnesia and was unable to remember family	NR	EEG indicated marked dysrhythmia, especially over the left frontotemporal region	NR	NR
Cermak 1976	44	Male	Malaise, fever and a headache. He then showed a language disturbance described as incoherent speech and amnesia.	NR	NR	NR	Discharged six weeks later
Yang 2015	37	Male	Behavioral abnormalities, confusion and amnesia	Negative	Brain MRI without contrast showed abnormalities. EEG on admission showed moderate (2–6.5 Hz) in the left fronto-temporal region without epileptic discharges	Acyclovir and prednisolone	Discharged one month later
Andrewes 1999	25	Female	Mild partial seizures, fever, headache, lethargy and poor recall for new verbal material.	NR	MRI scan results indicated evidence of damage to both left and right hippocampi and amygdalae. Specifically, there was high signal intensity in the posterior part of the left hippocampus and similar activity in the anterior aspect of both hippocampi	Acyclovir, penicillin, and phenytoin	Discharged then deteriorated markedly over the following five days, becoming amnesic
Fukushima 1986	36	Male	high fever and a headache which were followed by a tonic-clonic convulsion and the loss of Consciousness. Then he showed marked amnesia	Positive	Low density areas on the medial sides of both temporal lobes, basal forebrain, orbital gyri of the frontal lobes, and the insula gyri [CT]. Low intensity areas on the medial side of the right temporal lobe and the basal forebrain [MRI].	NR	He was diagnosed to have Retrograde Amnesia
Hori 1990	64	Male	Euphoria, flattening of affect and recent memory disturbance	NR	Brain CT and SPEW revealed nothing in particular. No abnormal findings were presented by an EEG examination	NR	He was discharged and followed up at the outpatient clinic
Midi 2007	37	Male	Severe headache of one-week duration that did not respond to analgesics. He could not remember words after a short period of time	Positive	MRI performed on the first day of admission revealed a hyperintense lesion in the right temporal lobe, with brain edema on T2- weighted images	Acyclovir	Sixth post-operative day, he was discharged and followed for one month as an in-patient
Tsukiura 2003	52	Female	Severe headache, fever, and severe anterograde and retrograde amnesia	NR	MRI revealed high intensity areas in the bilateral hippocampi, parahippocampal gyri, fusiform gyri, medial temporal poles, posterior part of the cingulate gyri, and insula. EEG showed normal basic rhythm and no epileptic discharge	NR	NR
Schmidt 1995	16	Female	Flu-like symptoms disoriented and becamed lethargic with difficulty with memory	Negative	CT showed right frontal hypo-densities. MRI reported high density in the temporal lobes, left more than right, with involvement of the left occipitoparietal lobe, right occipital lobe, and bilateral superior frontal cortices.	Acyclovir and ceftriaxone	Her fever decreased to low-grade and she was started on a rehabilitation regimen. She was discharged to her parents’ care after completing a twenty-one-day trial of acyclovir.
Kapur 1999	45	Male	Acute memory loss for recent events, an odd sense of smell, and episodes of de´ja` vu	Negative	CT scan on the day of admission was reported as normal, as was a routine MRI scan carried out 11. MRI one year later gave detailed views of memory-related brain structures days after admission. EEG investigations around this time showed bilateral temporal lobe abnormality	NR	NR
	35	Male	His symptoms included a marked impairment of recent memory that initially had some elements of confabulation.	NR	MRI showed discrete bilateral lesions in the area of the medial temporal lobes	NR	His condition deteriorated, and he died one month later

## Discussion

Viral encephalitis is a serious condition characterized by brain inflammation that can lead to neurological symptoms, including memory impairment. HSV-1 encephalitis is considered the commonest cause of sporadic fatal encephalitis globally, even with available antiviral treatment [29]. HSVE patients are classically present with fever, headache, and seizures. Over 90 % of adults exhibit classic signs and symptoms [30].

Viral encephalitis is commonly accompanied by changes in CSF composition, such as increased cellularity due to infiltration of immune cells and elevated protein levels [3]. Changes in CSF composition, such as increased cellularity brought on by immune cell infiltration and elevated protein levels, are frequently linked to viral encephalitis. However, our case report involves an unusual presentation where the CSF analysis revealed a hypocellular profile, considered a rare finding. This unusual finding could have some proposed explanations. First, it is possible that the CSF sample was taken at a time that was not the best for identifying inflammation. When CSF is collected in some viral encephalitis cases, the immune system might not have been fully activated. As a result, performing another lumbar puncture at a different time may produce a different set of results. However, the CSF sample was done three days after the beginning of symptoms, which is a sufficient period to cause the elevation of mononuclear cells, as mentioned in Ekmekci et al. [31].

Another explanation of this finding is that HSV has developed mechanisms to evade or suppress the immune system, resulting in atypical CSF findings [32], [33]. The inconsistency between our case's MRI findings and the CSF analysis raises several crucial concerns. Firstly, it points out the limitations of depending entirely on MRI results for diagnosis. MRI is useful for identifying structural abnormalities, but it may not always be consistent with CSF findings or clinical symptoms. As a result, clinicians interpret MRI results considering other diagnostic modalities. Secondly, our case emphasizes the importance of conducting thorough laboratory investigations in cases with contradictory findings. On the other hand, It is widely recognized from past studies that normal cerebrospinal fluid (CSF) cell counts can occur in HSV encephalitis, and it is recommended to perform repeated lumbar punctures in cases of suspicion. Also, normocellular CSF in HSE is not rare, and can be seen in normal as well as immunocompromised hosts as discussed in the study of Saraya et al. [34].

In the context of HSE, several case reports have highlighted different aspects of memory loss [10], [28]. As presented in our case, anterograde amnesia involving both verbal and visuospatial memory has previously been indicated in patients with HSE who have temporal lesions [15], [28], [35]. Eslinger et al. [28] described a 17-year-old girl with initial flu-like symptoms that progressed to agitation and photophobia followed by seizures and right hemiparesis. CT scan and T1-weighted MR images showed a well-defined abnormal signal area in the right hemisphere. The entire right temporal lobe was damaged except the posterior sector, the superior temporal gyrus. The patient exhibited marked retrograde and antrograde amnesia with significant dissociation between verbal and non-verbal learning. Previous studies [36] demonstrated non-verbal learning impairment in right medial temporal lobe surgery for epilepsy. Eslinger et al. [28] case affection of this area was severe and seemed to be associated with the destruction of the right medial temporal lobe.

Similarly, Sellner et al. [10] presented a 25-year-old pregnant woman who initially developed nonspecific symptoms such as fever, headache, nausea, and vomiting. However, as the disease progressed, the patient developed prominent memory deficits characterized by anterograde and retrograde amnesia. During a brain MRI scan, temporopolar and mesial hyperintensity was discovered in the right hemisphere using coronal fluid-attenuated inversion recovery (FLAIR) sequences. According to previous studies [37], damage to the dominant mesial temporal lobe (MTL) (usually the left) impairs verbal memory, while damage to the nondominant MTL (usually the right) impairs visuospatial memory [37]. However, memory affection is not a common presentation of acute HSV-1 encephalitis. It has mostly occurred post-infection as sequelae of the disease or secondary to immune-mediated mechanisms due to reduced white matter integrity [38].

## Conclusion

In contrast to typical MRI findings, this case report highlights a rare presentation of acute memory affection due to viral encephalitis with hypocellular CSF. The disparity between clinical symptoms, imaging results, and CSF analysis complicates diagnosis. Given the fatality of HSVE, it is critical to begin specific antiviral treatment as soon as a case is suspected to avoid complications and improve outcomes.

## Ethical approval

Not applicable.

## Conflict of interest statement

The author has no conflicts of interest to disclose.

## Consent

Written informed consent was obtained from the patient for the publication of this case report and accompanying images.

## Funding

None.

## CRediT authorship contribution statement

**Mostafa Meshref:** Writing – original draft, Validation, Methodology, Investigation, Formal analysis, Data curation, Conceptualization. **Abdullah Ashraf Hamad:** Validation, Methodology, Formal analysis, Data curation, Conceptualization. **Amira Mohammed Taha:** Validation, Resources, Methodology, Formal analysis, Data curation. **Yahia Nabil:** Writing – review & editing, Validation, Methodology. **Ahmed Hassan:** Supervision, Data curation. **Ahmed Samir Farw:** Supervision, Conceptualization. **Mohamed Elmasry:** Validation, Data curation, Conceptualization. **Abdulqadir J. Nashwan:** Writing – review & editing, Supervision, Conceptualization.

## Declaration of Competing Interest

The authors declare that they have no known competing financial interests or personal relationships that could have appeared to influence the work reported in this paper.
